# The “*Journal of Functional Morphology and Kinesiology*” Journal Club Series: PhysioMechanics of Human Locomotion

**DOI:** 10.3390/jfmk5030052

**Published:** 2020-07-18

**Authors:** Grazia Maugeri, Velia D’Agata, Federico Roggio, Cristina Cortis, Andrea Fusco, Carl Foster, Mark M. Mañago, Michael O. Harris-Love, Veronica Vleck, Maria Francesca Piacentini, Giuseppe Musumeci

**Affiliations:** 1 Department of Biomedical and Biotechnological Sciences, Anatomy, Histology and Movement Sciences Section, School of Medicine, University of Catania, Via S. Sofia 87, 95123 Catania, Italy; graziamaugeri@unict.it (G.M.); vdagata@unict.it (V.D.); federicoroggio@gmail.com (F.R.); 2 Department of Human Sciences, Society and Health, University of Cassino and Lazio Meridionale, 03043 Cassino, Italy; c.cortis@unicas.it (C.C.); andrea.fusco@unicas.it (A.F.); 3 Department of Exercise and Sport Science, University of Wisconsin-La Crosse, La Crosse, WI 54601, USA; cfoster@uwlax.edu; 4 Physical Therapy Program, Department of Physical Medicine and Rehabilitation, University of Colorado School of Medicine, Aurora, CO 80045, USA; manago@cuanschutz.edu (M.M.M.); michael.harris-love@cuanschutz.edu (M.O.H.-L.); 5 Geriatric Research, Education and Clinical Center, Rocky Mountain Regional Veterans Affairs Medical Center, Aurora, CO 80045, USA; 6 CIPER, Faculdade de Motricidade Humana, University of Lisbon, 1499-002 Lisbon, Portugal; veronica.triatlo@gmail.com; 7 Department of Movement, Human and Health Sciences, University of Rome “Foro Italico”, 00135 Rome, Italy; mariafrancesca.piacentini@uniroma4.it; 8 Research Center on Motor Activities (CRAM), University of Catania, 95123 Catania, Italy; 9 Department of Biology, Sbarro Institute for Cancer Research and Molecular Medicine, College of Science and Technology, Temple University, Philadelphia, PA 19122, USA

**Keywords:** human locomotion, PhysioMechanics, amyotrophic lateral sclerosis, nordic walking, multiple sclerosis, monitor training, gravity conditions

## Abstract

We are glad to introduce the Third Journal Club of Volume five, the third issue. This edition is focused on relevant studies published in the last years in the field of PhysioMechanics of Human Locomotion, chosen by our Editorial Board members and their colleagues. We hope to stimulate your curiosity in this field and to share with you the passion for the Sports Medicine and Movement Sciences seen also from the scientific point of view. The Editorial Board members wish you an inspiring lecture.

## 1. Introduction

Human locomotion can be considered the first archaic movement that distinguishes humans from animals. Three million years ago, the posture of humanoids started to change, the pelvis had been transformed from one adapted to quadrupedality to one tailored to terrestrial bipedality. One of the earliest adjustments to habitual bipedality in our ancestors was an elongation of the lumbar spine and the appearance of the lumbar lordosis that permitted the repositioning of the upper body over fully extended lower limbs. During the history many scientists studied the human locomotion, from the Edwin Smith Papyrus (1500 years BC) to Aristotle, Leonardo Da Vinci, Galileo or Newton, they were also interested in the analysis of normal and pathological movement and gait. Until the last century, walking was generally considered an automatic process involving little or no higher cognitive input. Nevertheless, reports of the past decades demonstrate that usual walking is controlled and defined by higher cognitive processes involving a complex neural network that incorporates sensory information with motor adaptation even to achieve everyday life tasks. Research is still investigating the indefinite number of elements that can be collected from the human movements useful to mark deviations from normal behaviour that suggest an underlying pathology. The analysis of human locomotion allows researchers to investigate how the impact of different pathologies, e.g., musculoskeletal, metabolic, cardiovascular, or neurological diseases may affect the health of humans. The lower cost of the intervention and the feasibility of training based on the locomotion, allow clinicians to act broadly both from a diagnostic and application point of view. Many types of interventions are widely diffused to treat or prevent some pathologies, for instance, the World Health Organization recommends at least half an hour of walking each day to reduce the incidence of diabetes by more than one half. From the treatment of lower limb osteoarthritis to the rehabilitation of spinal cord injuries with hypo-gravity training, from cardiovascular improvements due to Nordic Walking to the clinical evidence of exercise benefits for patients with stroke or multiple sclerosis, the human locomotion is increasingly understood as a valid and effective support tool in both diagnosis and treatment of different pathologies. Nevertheless, the study of human locomotion is crossing also with the future vision of human health supported by the help of robotic engineering. Bioengineering researchers are paying attention to the possibility to replicate human locomotion even with robotic technologies; for instance, the use of exoskeleton technology designed for people with paralyzed or weakened limbs for facilitating standing, walking, climbing stairs, and performing activities of daily living. Human locomotion is and will keep being a rich source of elements useful for prevention, diagnosis, treatment, and support of many pathologies.

## 2. Locomotion Analysis and Its Applications in Amyotrophic Lateral Sclerosis

### Highlight by Grazia Maugeri and Velia D’Agata

Human locomotion represents one of the main features that widely involves both motor ability and adaptability. Walking on our two feet is likely the most frequently used mode of locomotion as well as the most practiced physical activity in the world. This locomotor skill is acquired from one year old in age and maintained throughout life. Nevertheless, walking is a very complex activity requiring the intervention of both cortical and subcortical structures, and most of the muscles of the human body. Alteration in locomotion occurs in different pathologies including amyotrophic lateral sclerosis (ALS). The ALS is a degenerative, progressive, and paralytic disorder targeting motor neurons (MNs) of the brain and spinal cord [1]. MNs degeneration leads to walking alteration, spasticity, uncontrolled movement, significant reduction of sensitivity, muscular atrophy and, paralysis in the end-stage [2,3]. The mechanisms underlying MNs death susceptibility are not fully understood. Previous studies have suggested that environmental factors, such as exposures to agriculture chemicals, solvents, heavy metals, electrical magnetic fields, physical activity, and type of diet may represent risk factors to develop ALS [4,5,6]. The early detection of ALS is a current challenge since symptoms are similar to other motor disorders (e.g., multifocal motor neuropathy, myasthenia gravis, and the inflammatory myopathies). The diagnosis of ALS is usually based on the examination of patient history and different clinical tests, comprising electrodiagnostic, neuroimaging studies, and genetic testing [7,8,9]. However, these approaches cannot achieve an early and reliable detection. In this *scenario*, locomotion analysis, with the identification and interpretation of minor gait abnormalities, may be helpful for early detection of ALS. Over the past few years, different methods have been developed for the locomotion analysis [10,11,12]. In particular, Tang et al. [13] introduced a new methodology able to capture locomotion deficiencies by measuring parameters in four domains: Force, distance, time, and frequency. By applying this method to G93A-superoxide dismutase 1 (SOD1) transgenic rats, it has been possible to detect motor deficits similar to that observed in ALS with specificity and sensitivity values of at least 90%. Therefore, identification of novel and sophisticated locomotion analysis methods may represent a valid mean for the early detection of diseases affecting motricity.

## 3. Nordic Walking Benefits on Postural Control and Cardiovascular System

### Highlight by Federico Roggio

Nordic Walking (NW) is a walking-based physical activity supported by the use of a pair of poles in opposition to the lower limb locomotion that actively engages the upper body. Due to high muscle recruitment, NW compared to normal walking has proved to be effective in both maintenance and improvement of the cardiovascular system function. Pellegrini et al. investigated how the use of poles affects the mechanical energy needed in the locomotion pattern of the body centre of mass. The presence of poles involves the upper body to oscillate more during the gait, namely a higher mechanical work and energy expenditure, lower metabolic efficiency, isometric and muscle co-activation in the upper body [14,15,16]. The trunk response is significant as well because the muscle co-activation provides adequate spinal stability in different conditions, and it can be meaningful in order to improve the postural stability of the core/lumbopelvic region [17]. The activity of erector spinae longissimus and multifidus lasts for more portions of the gait cycle during the uphill NW to contrast the torque of the body longitudinal axis [18]. NW has shown benefits also for patients with coronary [19] and arterial diseases [20] because it does not produce significant stress on the heart. Girold et al. studied the effects on patients with acute coronary syndrome and peripheral arterial occlusive disease by administering a short-term intensive training program and testing them with a 6-min walk test before the protocol and then at the end of it. The outcomes showed significant improvement in distance covered on the 6MWT specifically for patients with the peripheral arterial occlusive disease [21]. To the clinician who considers NW an exercise prescription, a training program is recommended lasting from 8 to 12 weeks at least, two sessions per week, according to Gomeñuka et al. and Bullo et al. to ensure improvements on cardiovascular outcomes, muscle strength, balance ability, and quality of life [22,23], a shorter duration might produce no improvements.

## 4. Flat or High?

### Highlight by Cristina Cortis, Andrea Fusco, and Carl Foster

During everyday life, walking occurs with several types of footwear, ranging from the most comfortable, such as sandals, to the most fashionable, such as high-heeled shoes. Each requires different levels of intermuscular coordination and potentially energy cost [24]. Among sandals, flip-flops have rapidly increased in popularity due to lightweight, convenience, and comfort. Different peak ground reaction force variables, ankle and knee kinematic moments were shown during level-walking in flip-flops compared to running shoes [25] suggesting an impact of footwear on lower extremity mechanics. In particular, the higher loading rate of the peak ground reaction force in flip-flops could be attributed to the thin sole and the lack of cushioning material not being able to provide force attenuation capabilities, thus making flip-flops less than ideal during long distance walking. Significant differences in the ankle angle in a swing, frontal plane motion, and loading rate of the vertical ground reaction force have also emerged when flip-flop walking was compared to barefoot [26]. In flip-flops only the front part of the foot is covered, thus individuals need to adapt their gait to hold the shoe on the foot resulting in different kinetic and kinematic parameters of the gait cycle. During flip-flop walking significantly lower walking speed, a higher ankle and subtalar joint range of motion, and a higher shear ankle joint contact force, although no differences in co-contraction, were reported compared to sports shoes during 10-m at a self-selected walking speed [27]. The findings suggest that sports shoes with close-toe design should be preferred to constrain joint motion and loading especially in individuals who are not regular flip-flops wearers. However, flip-flops showed no alteration in walking pace or overall energy expenditure compared to other types of shoes [28] which means that the characteristics of the exercise (i.e., a one-mile walk at preferred pace) is not enough to elicit a significant modification in pacing strategy.

In contrast with flip-flops, high-heeled shoes represent the perfect love-hate relationship for each woman who wears them to look elegant, fashionable, and taller. They usually experience some discomfort, especially during prolonged walking. A single variation in gait patterns, such as increasing heel height, changes the whole pattern with the most significant changes occurring at ankle and knee joints [29], raising and shifting forward the center of mass, thus increasing the vertical loading. In particular, ankle and knee flexion, ground reaction and braking force, and energy cost increased when walking at a normally “comfortable” walking speed (4.2 km/hour) when wearing high-heeled shoes, especially with heel heights above 5.08 cm [30]. When walking in high-heeled shoes (heights ranging from 1 to 9 cm) the foot is lifted more, thus resulting in more effort, and greater acceleration occurs, thus also more attention must be paid at each step to prevent mis-steps and falling [31]. A recent review [29] highlights changes in the rollover function of the feet while step length and balance are compromised, suggesting individuals must improve their balance control to securely walk on high-heeled shoes. However, during balance evaluation, female individuals showed better performance than the male counterpart in the anterior direction of the Y balance test [32], attributable to the habit of using high heels. It is possible that women, thanks to high-heeled shoes indirect training, might have mastered specific ankle proprioception and movement, resulting in better balance than males, leaving this relation unsolved as a sort of chicken or egg causality dilemma.

## 5. Assessment of Ankle Plantarflexion Muscle Function in Patients with Multiple Sclerosis

### Highlight by Mark M. Mañago and Michael O. Harris-Love

Given that the lesions associated with multiple sclerosis (MS) may affect multiple brain and spinal cord areas, there are a wide variety of gait abnormalities associated with this neurological disease [33]. The ankle plantar flexors are a critical driver of gait for people with MS, particularly during push-off in the terminal stance period of the gait cycle [34]. Deficits in ankle plantarflexion muscle function (strength and endurance) are common in people with MS [35] and are associated with worse walking performance (speed and endurance) [36]. Therefore, improving ankle plantarflexion muscle function in people with MS is a common goal of clinical rehabilitation intervention [37]. However, measuring the performance of the ankle plantar flexors in a clinical setting can be challenging, as electromechanical or fixed dynamometry may not be feasible [38]. Furthermore, manual muscle testing and hand-held dynamometry may not be valid for measuring ankle plantarflexion strength, as the tester strength is typically insufficient to overcome the force of the ankle plantar flexors. These methods may be insufficient to assess strength even when there is significant muscle weakness at the ankle joint [39,40]. Therefore, protocols that test ankle plantar flexor muscle endurance via repeated calf-raises are commonly used in the clinical setting, despite the fact that they may not correlate particularly well with measures of ankle plantar flexor muscle strength as assessed by electromechanical or fixed dynamometry [39,41]. In people with MS, ankle plantarflexion endurance measured by repeated calf raises [42] and ankle plantarflexion strength measured by electromechanical dynamometry [43] have both been shown to correlate strongly with walking performance. Both measures have also been shown to differentiate between people with MS and a healthy control group [34,42]. Thus, while the calf-raise test and dynamometry measure different constructs of ankle plantarflexion muscle function, both appear to be valid assessments in people with MS. In addition, the calf-raise test has been shown to have high reliability in people with MS [34]. Recently published normative reference data [44] supports the reliability of the calf-raise test with mean inter-session differences in total repetitions of 0.2 with a standard error of measurement of 2.2 repetitions (95% confidence limits: 1.6, 3.2). Therefore, the clinically feasible calf-raise test may be an appropriate method to identify deficits in ankle plantarflexion muscle function in people with MS and help clinicians make decisions about intervention targets and outcomes. Future research should continue to investigate the psychometric properties of the calf-raise test in patient populations and validate clinically feasible ankle plantarflexion muscle function assessments in people with MS and other neurological conditions.

## 6. Objective or Subjective Markers to Monitor Training during Water Locomotion or Ground Locomotion?

### Highlight by Veronica Vleck and Maria Francesca Piacentini

The need for training loads to be administered in a logical manner, so as to promote positive adaptation and prevent the development of non-functional overreaching (NFOR) or overtraining syndrome (OTS), implies a systematic and well-planned approach to the development of a training programme [45]. Prolonged training and insufficient recovery, together with other stressors such as training monotony, excessive competition, personal and emotional (psychological) problems, and occupational stress may engender chronic maladaptations that can lead to OTS [46].

For training regulation to be optimized, it is important that both the training load, and how an athlete is responding to the training process, be accurately assessed. Multiple methods of monitoring training, assessing various aspects of training adaptation, and/or training related fatigue, exist. Research has concentrated on identifying the most important subjective markers (such as session RPE, physical and mental wellbeing, sleep monitoring), that can actually help a coach understand whether a planned overload (training camp, altitude camp) will elicit NFOR instead of functional overreaching (FOR) [47]. But are there differences in what we monitor based on mode of locomotion? Just recently, Ieno et al. [48] reported that the sRPE breakpoints to monitor training intensity distribution in open water swimmers differ from what has been reported by Seiler [49] for cross country skiers and terrestrial sports in general. This is due to the high level of technical ability that is necessary to succeed in swimming and to the higher energy cost of water-based locomotion.

It is also well documented that athletes manifesting signs of OTS and NFOR may exhibit immune, inflammatory, neurological, hormonal, and/or metabolic system pathway and response dysfunction [50], justifying the tracking of the mood disturbances that are characteristic of such maladaptation [47]. Meeusen et al. [51] demonstrated a dysfunctional hypothalamic-pituitary-adrenal (HPA) axis response to exercise, resulting in an altered hormonal response to intense training and competition. For this reason, biochemical monitoring has been suggested as a means by which the coach may better comprehend the training (mal)adaptations of an athlete. The tracking of adipokine levels in addition to those of more commonly assessed hormonal markers such as testosterone and cortisol may provide insight in this regard [52]. It may aid, for example, in assessing which of several periodization models may be most appropriate in a given situation. Hornsby et al.’s 2020 descriptive study explored both how adipokine concentrations may change with training, and their potential use as a monitoring tool, in a real-world athlete monitoring related setting. Although the investigation was purely exploratory, the study is interesting because of its high ecological validity and the detail to which it reported the athletes’ training. The authors state that “including the inflammatory-related hormone response” (in the monitoring battery) “may be another helpful piece of the overall athlete response puzzle”. 

Although biochemical testing may be impractical, and is incapable of detecting NFOR early enough, it may be useful in divining the athlete’s actual health status [46]. We note that although knowledge of the central pathomechanisms of OTS has significantly increased in recent years, the continued strong demand for tools that are relevant to the early diagnosis of OTS appears largely to focus on subjective monitoring methods [46]. The possibility that inherent variation in sRPE values exists across locomotory modes potentially introduces an added level of complexity to its use for training monitoring- with a view to performance optimization- in multi-disciplinary sports such as the triathlon.

## 7. Human Locomotion under Reduced Gravity Conditions

### Highlight by Giuseppe Musumeci

The force of inertia, that is, the ability of the body to resist a change of motion, is summarized in Newton’s First Law and exemplified by centrifugal force. “Everybody continues in its state of rest or uniform motion in a straight line except in so far as it is compelled by forces to change that state”. In other words, if an object is not experiencing the action of an external force it will either keep moving or not move at all. This law expresses the concept of inertia, the inertia of a body can be described as being its reluctance to start moving or stop moving once it has started. Immobility is one of the most common risk factors that increase the risk to develop relevant non-communicable diseases and their related risk of mortality [53,54]. The longtime immobility causes alterations of the tissues associated with joint motion such as bones, cartilage, muscles and ligamentous elements [55]. Stiffness and viscosity are physical properties that change with muscle and joint functional adaptations [56,57,58,59]. To counteract and contain this phenomenon, worldwide actions aimed to promote specific health prevention interventions. These interventions are addressed to reduce cardiometabolic risk factors, combining a balanced diet with an adequate level of weekly physical activity [60]. For the above reasons, walking can be performed in different environments with no need of particular equipment, overcoming some common barriers such as the lack of time, low fitness level, and shortage of money. Recently the scientific community focused its attention on hypogravity training/rehabilitation, in fact, reduced gravity is a good strategy for learning and understanding motor behavior and locomotion in human. In an interesting and recent paper by Francesca Sylos-Labini et al., the authors aimed to provide an overview of current issues of the known tools and techniques used for hypogravity simulation and their effects on human locomotion [61]. Walking and running rely on the limb oscillatory mechanics, and one way to change its dynamic stuff is to modify the level of gravity. Gravity has a solid outcome on the optimal rate of limb oscillations, optimal walking speed, and muscle activity patterns, and gait transitions occur smoothly and at slower speeds at lower gravity levels. Distorted centre of mass movements and interplay between stance and swing leg dynamics may test new methods of locomotion in a hypogravity setting [62]. Moreover, annotations in the lack of gravity effects help reveal the intrinsic properties of locomotor pattern generators and make marked facilitation of non-voluntary limb stepping. Reduced gravity additionally suggests exclusive chances for adjusting the basic patterns to altered locomotor conditions for gait rehabilitation. Bodyweight support systems coupled with robotic devices or pharmacologic behaviours are often expended in the rehabilitation practice to assist physical therapy of persons with neurological disorders. New pharmacological treatments [61] and electromagnetic stimulation techniques [62,63,64] are being developed aimed at modulating spinal activity and restoring the locomotor function. The spinal central pattern generator (CPG) circuitry can be simply activated in healthy humans in a gravity neutral position by applying tonic central and peripheral sensory inputs. To reduce interfering with the ongoing task of body weight and balance control, stepping movements are elicited during air-stepping in the lack of gravity influences and external resistance. This viewpoint outlines an interdisciplinary approach to extend our knowledge on the adaptation of human locomotion to a hypogravity environment, including biomechanical, neurophysiological, and comparative features. The tools and techniques used for hypogravity simulation and their consequences on human locomotion provide new insights into our understanding of the physiological effects of gravity. The beneficial effect of weightlessness on rhythmogenesis would further enhance the usefulness of this methodology and growth of state-of-the-art knowledge for gait rehabilitation.
